# Quantification of daily upper arm use after stroke using an IMU-based kinematic model

**DOI:** 10.3389/fneur.2026.1860647

**Published:** 2026-08-06

**Authors:** Noy Goldhamer, Yogev Koren, Tamar Mizrahi, Adi Lorber-Haddad, Reut Binyamin-Netser, Lior Shmuelof

**Affiliations:** 1 The Lillian and David E. Feldman Research Center for Rehabilitation Sciences, Adi Negev - Nahalat Eran Medical Center, Ofakim, Israel; 2 Department of Industrial Engineering and Management, Ben-Gurion University of the Negev, Be’er Sheva, Israel; 3 Neurologic Rehabilitation Department at ADI Negev-Nahalat Eran, Ofakim, Israel; 4 Department of Occupational Therapy, Ben-Gurion University of the Negev, Be’er Sheva, Israel; 5 The School of Brain Sciences and Cognition, Ben-Gurion University of the Negev, Be’er Sheva, Israel

**Keywords:** activity asymmetry, ecological validity, motor performance monitoring, stroke rehabilitation, upper limb function, wearable motion sensors

## Abstract

**Background:**

Upper limb (UL) impairment is one of the most common and disabling consequences of stroke, limiting independence and the performance of daily activities. Although rehabilitation aims to reduce impairments and enhance real-world activity performance, clinical motor assessments often fail to capture spontaneous UL use. This discrepancy between motor capacity observed in clinical settings and actual performance of daily activities underscores the need for objective, ecologically valid monitoring tools. Wearable inertial measurement units (IMUs) have emerged as feasible methodology for quantifying real-world UL use; however, the clinical interpretability and validity of kinematic metrics derived from measures of activity in persons with stroke (PwS) remain insufficiently established.

**Objective:**

To evaluate the utility of three IMU-derived kinematic measures of UL use in persons with stroke (PwS) during routine daily activity and to examine their associations with clinical assessments of motor impairment.

**Methods:**

Nineteen individuals in the subacute stage after stroke wore five IMU sensors during routine daytime activities in a rehabilitation hospital. Three kinematic measures - wrist path length, cumulative elbow angular displacement, and movement space volume - were extracted for each arm and combined to asymmetry indices. Clinical assessments included the Fugl-Meyer Assessment for the upper extremity (FMA-UE), Action Research Arm Test (ARAT), and grip strength. Asymmetry indices and their correlations with clinical measures were analyzed.

**Results:**

All IMU-derived measures were significantly reduced in the paretic arm compared with the non-paretic arm (*p* < 0.001). Asymmetry indices showed significant correlations with clinical impairment measures, with cumulative elbow angular displacement demonstrating the strongest association, particularly with ARAT scores.

**Conclusion:**

IMU-based kinematic measures effectively capture reduced daily UL use in PwS and demonstrate associations with clinical impairment. These findings support the potential of wearable sensors to quantify daily arm use as an ecologically valid indicator of activity performance and recovery.

## Background

Stroke is a leading cause of long-term disability worldwide, with upper limb (UL) impairment among its most prevalent and functionally disabling consequences. Approximately 77% of persons with stroke experience upper-limb weakness in the acute phase (1). Upper-limb impairment contributes substantially to functional disability after stroke and limits independence in activities of daily living (ADL) such as feeding and grooming (2). Motor rehabilitation after stroke aims to improve motor function, activity capacity and performance in daily life through learning- and use-dependent mechanisms. In clinical practice, treatment is typically designed to reduce impairment levels, based on the assumption that such reduction will naturally translate into reduced disability, enhanced functional ability, and increased use of the affected arm in daily life. That is, clinicians often expect that regained motor capacity observed in the clinic will generalize to everyday behavior. This assumption is supported, to some extent, by observed associations between impairment severity and self-reported measures of activity and participation (3). However, accumulating evidence indicates that such self-reported assessments often fail to reflect how much PwS use their paretic arm in daily life (4). Clinical test scores do not fully capture spontaneous, context-dependent UL use, particularly in unsupervised, real-world settings (5). For example, the ability to reach for, grasp., and drink from a glass in a clinical setting does not necessarily translate into consistent use of the affected arm at home, nor does it guarantee that the same performance can be reproduced in more complex or distracting environments.

This discrepancy challenges a fundamental assumption underlying many rehabilitation approaches. This gap is commonly framed as the distinction between activity capacity - what an individual can do under standardized conditions - and activity performance - what the individual does in the usual environment (4, 6–8).

Several studies have shown that PwS frequently use their paretic arm less than would be predicted based on their level of impairment (4, 9). This behavioral pattern, commonly referred to as learned non-use, may persist unless explicitly addressed in therapy (10). Learned non-use is detrimental because it reinforces compensatory strategies, constrains recovery, and ultimately reduces the affected limb’s engagement in daily activities. Nevertheless, monitoring and quantifying intentional, goal-directed UL movements in daily life remains challenging.

In recent years, wearable sensors have gained increasing attention as tools for capturing and quantifying both lower-limb and upper-limb impairment (11–13). Inertial measurement units (IMUs), which integrate accelerometers, gyroscopes, and magnetometers are particularly well suited for this purpose because they provide high temporal resolution, are lightweight and wireless, and enable prolonged monitoring in naturalistic environments. While IMU-based assessment of motor impairment after stroke has been demonstrated [see a recent review in (13)], and the feasibility and usability of IMU systems for continuous monitoring of daily activity was shown (14) their utility in assessing the difference between capability and real-world activity performance remains in its infancy. Furthermore, UL activity is often quantified as the ratio of activity time between the paretic and non-paretic arms to account for large variability between individuals, but the specific parameters used to quantify arm use vary substantially across studies. As a result, it remains unclear which metrics are most informative for capturing meaningful aspects of daily UL use (11, 15). The aim of this pilot study is to demonstrate the potential utility of three IMU-derived outcome measures of daily UL use. These measures were obtained from PwS during their hospitalization in a rehabilitation hospital to provide complementary insights into real-world UL use that extend beyond what traditional clinical assessments capture.

## Materials and methods

The study was conducted at the Lillian and David E. Feldman Research Center for Rehabilitation Sciences, Adi Negev – Nahalat Eran rehabilitation hospital. Research protocols were approved by the local Ethical Committee (ADINEGEV-2023_106), and all participants provided written informed consent.

### Participants

Nineteen individuals in the subacute stage after stroke participated in the study. Inclusion criteria were: (1) unilateral UL hemiparesis following stroke, and (2) capacity to provide informed consent. Participants were excluded if they had additional neurological or orthopedic conditions that could interfere with movement. Descriptive demographic and clinical characteristics are presented in Table 1. All participants reported right-hand dominance prior to the stroke. Dominant arm was affected in 11/8 of the participants (Table 1). The relatively narrow ranges of age and time since stroke reflect the characteristics of the inpatient rehabilitation population from which participants were recruited.

**Table 1:** Participant characteristics (mean ± SD).

Characteristic	Value
Age, years	66.1 ± 8.3
Sex (male/female), *n*	13/6
Weight, kg	75.2 ± 12.6
Affected side (Right/Left)	11/8
Time after stroke onset, days	51 ± 23.4
Fugl-Meyer upper extremity (0–66)	35.2 ± 20.6
ARAT (0–57)	24.7 ± 21.3
Grip, normalized to age and sex (z-score)	−3.2 ± 1.7

### Experimental procedure

**Figure 1:**
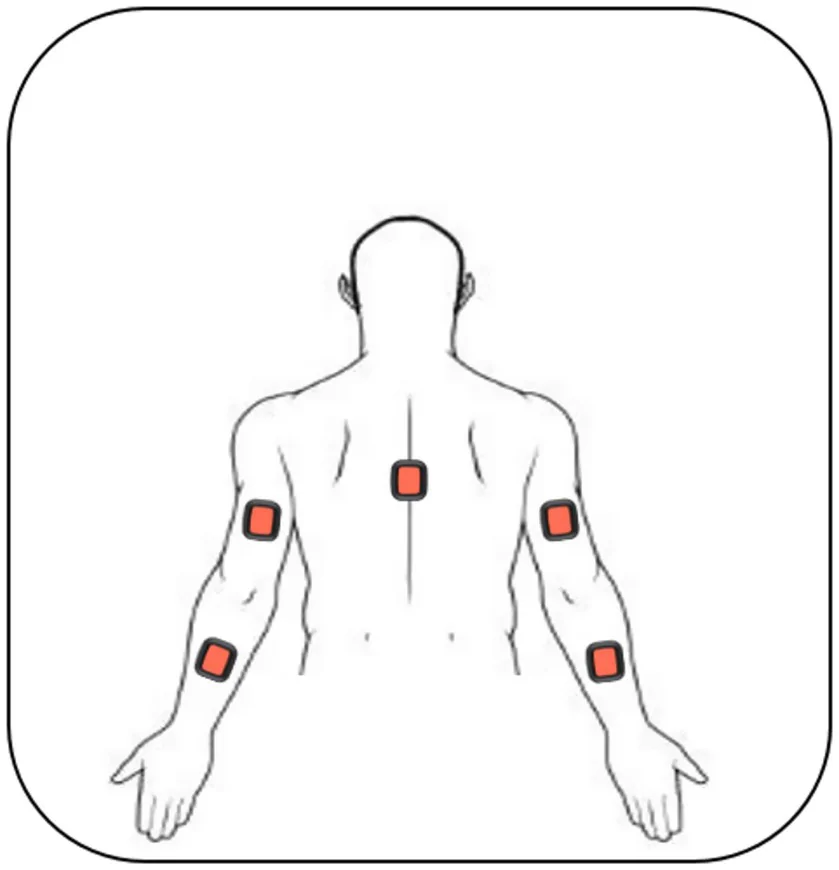
Sensor placement.

Daily upper-limb activity was monitored using five Xsens DOT inertial measurement units (Movella, Henderson, NV, United States). One sensor was attached to each upper arm, just below the shoulder, one to each distal forearm proximal to the wrist, and one to the posterior torso between the scapulae (Figure 1). The sensors were secured directly to the body using kinesiological tape and were placed in a consistent orientation across participants. Before each recording, the five sensors were synchronized using the Movella DOT App.

Each participant completed one recording session during a routine weekday in the inpatient rehabilitation hospital. Sensor placement occurred between 08:30 and 11:00, and sensors were removed between 14:00 and 16:00. Participants continued their usual prescheduled rehabilitation program and daily routine while wearing the sensors. Hydrotherapy was not performed during the recording because the sensors were not waterproof. Activities were not logged or classified.

The sensors recorded three-dimensional linear acceleration, angular velocity, and magnetic-field data at 60 Hz. The sensors directly provided orientation quaternions calculated by the manufacturer’s proprietary onboard sensor-fusion algorithm, which incorporates accelerometer, gyroscope, and magnetometer signals. No additional temporal filtering, magnetometer-independent orientation estimation, or drift-correction procedure were applied. For calibration, participants were instructed to stand upright for 10 s with both arms extended alongside the body. The upper-arm and forearm sensors were positioned so that their longitudinal axes were aligned vertically during this posture. For each sensor, the mean orientation quaternion over an approximately 8-s stable segment of the initial calibration posture was used as the reference orientation. Before recording, participant-specific anthropometric measurements were obtained using a measuring tape. These included upper-arm length from the shoulder to the elbow, forearm length from the elbow to the wrist, back length from shoulder level to the waist, and shoulder width measured between the left and right acromial landmarks. All measurements were entered into the participant-specific kinematic model.

The analyzable recording interval was restricted to the time period during which valid data were concurrently available from all five sensors. If the battery of one sensor was depleted before the others, the recording was truncated at the last sample shared by all sensors. Data recorded by the remaining sensors after that point were excluded. No complete recording was excluded because of missing sensor data. At the end of each session, sensor placement was visually inspected, and no apparent displacement requiring data exclusion was identified. The calibration procedure was not repeated at the end of the recording.

### Clinical measures

Motor impairment was evaluated by a trained clinician using three standardized assessments: Fugl-Meyer Assessment for Upper Extremity (FMA-UE) (16): scored 0–66; lower scores indicate greater impairment, Action Research Arm Test (ARAT) (17): scored 0–57; lower scores indicate greater impairment and Grip Strength (GS) measured with a digital dynamometer (microFET HandGRIP). Force values were normalized for age, sex, and hand dominance (18).

### Kinematic modeling

Joint kinematics were estimated from the orientation quaternions provided directly by the Xsens DOT sensors. The quaternions were generated by the manufacturer’s proprietary onboard sensor-fusion algorithm using accelerometer, gyroscope, and magnetometer signals; orientation was therefore not derived from acceleration data alone. Sensor timestamps were corrected for occasional timestamp resets occurring during prolonged recordings and aligned to a common 60-Hz time vector. Kinematic reconstruction was performed only for samples for which valid quaternion data were simultaneously available from all five sensors. For each sensor, orientation was expressed relative to its own mean orientation during the initial calibration posture. At each subsequent sample, the relative orientation was calculated as:

$$q_{s,\mathrm{rel}}(t)=q_{s,\mathrm{cal}}^{-1}⊗q_{s}(t)$$

Where q_s_ (t) is the current orientation quaternion of sensor s, q_s,cal_ is its mean calibration quaternion, and ⊗ denotes quaternion multiplication.

Initial upper-arm and forearm segment vectors were defined as vertically downward during the calibration posture, whereas the trunk vector was defined as vertically upward. At each sample, these initial vectors were rotated according to the corresponding sensor orientation relative to calibration and scaled using the participant-specific segment lengths.

Shoulder, elbow, and wrist positions were reconstructed using a rigid-segment forward-kinematic model. The elbow position was calculated by adding the rotated upper-arm vector to the corresponding shoulder position:

$$P_{\text{elbow}}(t)=P_{\text{shoulder}}(t)+r_{\text{upper}\;\mathrm{arm}}(t)$$

The wrist position was subsequently calculated by adding the rotated forearm vector to the reconstructed elbow position:

$$P_{\text{wrist}}(t)=P_{\text{elbow}}(t)+r_{\text{forearm}}(t)$$

The two shoulder positions were reconstructed using the trunk vector and the participant’s measured shoulder width. The model treated the upper arm, forearm, and trunk as rigid segments and did not include independent scapular or glenohumeral motions. Following position reconstruction, all joint trajectories were transformed into a body-centered coordinate system by subtracting the reconstructed displacement of the right shoulder from all joint positions. This procedure reduced the effect of whole-body translation but did not eliminate potential relative inter-sensor drift, magnetic disturbances, sensor-to-segment misalignment, or sensor slippage.

The elbow angle was calculated as the three-dimensional angle between the reconstructed upper-arm and forearm vectors:

$$\theta_{\text{elbow}}(t)=\text{atan}2(\|u(t)\times v(t)\|,u(t)\cdot v(t))$$

Where u(t) and v(t) represent the upper-arm and forearm vectors, respectively. The resulting angle was converted from radians to degrees.

### Quantities of daily use (participation)

Three IMU-derived kinematic measures were computed for each arm:

*Wrist path length*: The cumulative three-dimensional distance traveled by the reconstructed wrist was calculated as the sum of the Euclidean distances between consecutive samples:

$$D_{\text{wrist}}=\sum_{t=2}^{T}P_{\text{wrist}}(t)-P_{\text{wrist}}(t-1)2$$

Because segment lengths were entered in centimeters, the resulting cumulative distance was converted from centimeters to kilometers and normalized by the valid recording duration in hours. No temporal smoothing or minimum-displacement threshold was applied before calculating the cumulative path length.

*Cumulative elbow angular displacement*: The cumulative elbow angular displacement was calculated as the sum of the absolute frame-to-frame changes in the three-dimensional elbow angle:


$$\text{CAD elbow = }\sum_{t=2}^{T}|\theta_{\text{elbow}}(t)-\theta_{\text{elbow}}(t-1)|$$

The cumulative value was normalized by the valid recording duration and reported in degrees per hour. No temporal filtering or minimum angular-change threshold was applied.

#### Movement-space volume

Movement-space volume was calculated separately for each wrist from all valid reconstructed wrist positions. To reduce the influence of extreme position estimates, robust multivariate outlier rejection was first performed. A robust estimate of the mean and covariance was obtained using MATLAB’s *robustcov* function, and the squared robust Mahalanobis distance was calculated for each wrist-position sample. Samples exceeding the 97.5th percentile of a chi-square distribution with three degrees of freedom were excluded. The volume of the three-dimensional convex hull enclosing the remaining wrist positions was then calculated using MATLAB’s *convhulln* function. Unlike wrist path length and cumulative elbow angular displacement, movement-space volume was not normalized by recording duration. The resulting volume was converted from cubic centimeters to cubic meters.

Wrist path length and cumulative elbow angles were normalized by recording duration to account for variations among participants.

To quantify activity asymmetry while accounting for inter-individual differences in overall movement magnitude, each participant’s measures were converted to a normalized asymmetry index:

$$\text{Asymmetry}=\frac{\text{NonParetic}-\text{Paretic}}{\text{NonParetic}+\text{Paretic}}$$

Positive values indicate greater activity of the non-paretic arm, values near zero indicate symmetric activity, and negative values indicate greater activity of the paretic arm.

### Statistical analysis

Paired t-tests were used to compare kinematic measures between paretic and non-paretic arms. Pearson correlation coefficients assessed associations between activity asymmetry measures and clinical assessment scores. Confidence intervals for Pearson’s correlation coefficients (*R*) were determined via non-parametric bootstrapping using 1,000 resamples with the percentile method. All analyses were performed in (JASP, V.0.95.3). with a significance threshold set at *α* < 0.05.

## Results

Nineteen PwS were monitored during their hospitalization. The mean time after stroke onset was 51 ± 23.4 days, and the mean ± SD recording duration was 278 ± 36.5 min, with a range of 203–315 min. All participants were right-handed prior to stroke (Table 1).

**Figure 2:**
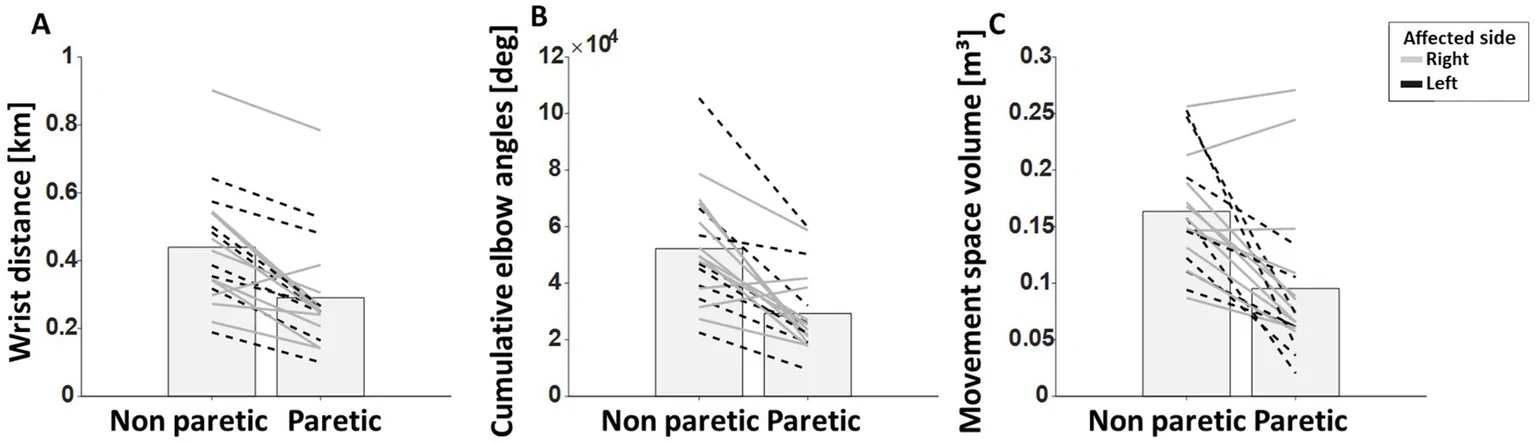
Comparison of wrist distance **(A)**, cumulative elbow angles **(B)**, and movement space volume **(C)**, between the non-paretic and paretic arms. Bars represent the mean group value, and each line represent the 2 values of each participant.

We first compared the three IMU-derived kinematic measures between the non-paretic and paretic arms. All measures showed significantly lower values for the paretic arm, indicating reduced use relative to the non-paretic arm (Figure 2 and Table 2).

**Table 2:** Kinematic measures in the non-paretic and paretic arms.

Mean ±SD	Non-Paretic	Paretic	*p*-value (paired *t*-test)
Wrist path length (km/h)	0.44 ± 0.164	0.30 ± 0.155	*p* < 0.001
Cumulative elbow angles (deg/h)	5.22 ± 1.938(×10^4^)	2.93 ± 1.378(×10^4^)	*p* < 0.001
Movement space volume (m^3^)	0.16 ± 0.05	0.10 ± 0.0636	*p* < 0.001

Interestingly, although most PwS demonstrated lower use of the paretic arm, a subset of PwS showed the opposite trend (Figure 2). A plausible explanation is that the paretic hand was also the dominant hand. Indeed, in all cases where the paretic arm showed higher activity, it was also the participant’s dominant arm (Figure 2). This suggests that, in some individuals, dominance may exert a stronger influence on real-world arm use than impairment. These findings support the validity of the IMU-derived measures as ecologically relevant indicators of spontaneous use in daily activities.

**Figure 3:**
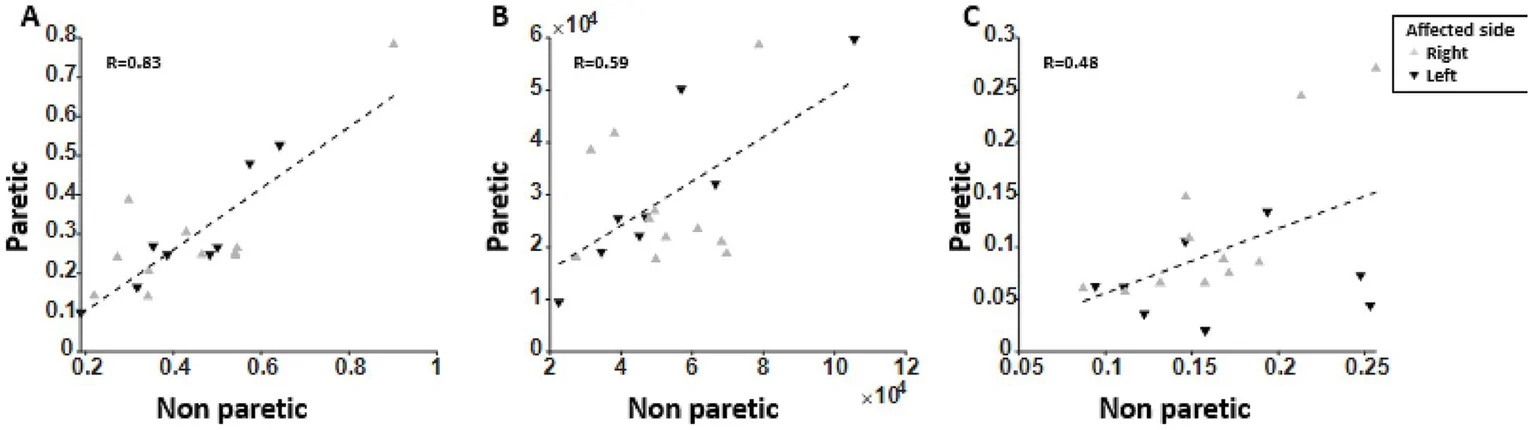
Correlations between the paretic and non-paretic arms for wrist distance **(A)**, cumulative elbow angles **(B)**, and movement space volume **(C)**.

Next, we explored the associations between the kinematic measures and clinical measures of motor impairment. Because absolute kinematic values varied across participants (with inter-arm correlations exceeding R = 0.48; Figure 3), participation measures were expressed as asymmetry indices. These normalized values quantify the relative contribution of the paretic arm, independent of interpersonal differences, in overall movement magnitude arising from impairment severity and learned non-use.

**Figure 4:**
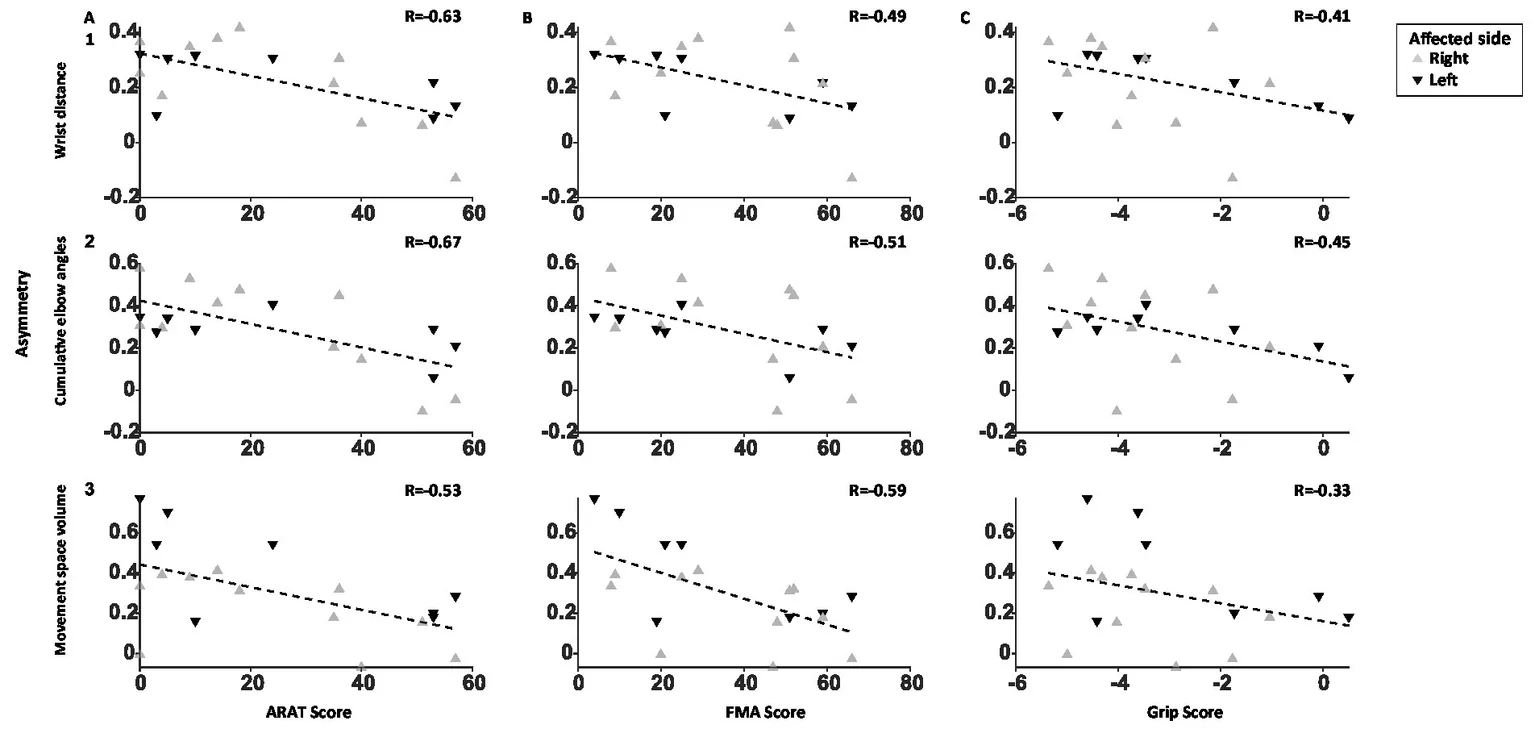
Correlations between wrist distance (first row, 1), elbow angle (second row, 2), and space volume (third row, 3), and the ARAT score (first column, **A**), FMA score (second column, **B**), and grip strength (third column, **C**).

Results of this analysis revealed a significant correlation between the three activity measures and clinical measures of impairment (Table 3; Figure 4). Among the kinematic variables, cumulative elbow angles showed the strongest correlations with clinical measures. Among clinical assessments, ARAT demonstrated the strongest correlations with activity measures. The associations of the activity measures with grip strength were the weakest.

**Table 3:** Correlations between asymmetry measures of daily use and clinical assessments.

Variable name	ARAT	FMA	Grip
Wrist distance	R = −0.63 (−0.85 - -0.25), *p* = 0.004, Fisher’s *z* = −0.74	*R* = −0.49 (−0.788 - -0.106), *p* = 0.03. Fisher’s *z* = −0.54	*R* = −0.47 (−0.76 - -0.036), *p* = 0.04. Fisher’s *z* = −0.51
Cumulative elbow angle	*R* = −0.67 (−0.84 - -0.38), *p* = 0.03. Fisher’s *z* = −0.81	*R* = −0.51 (−0.77 - -0.189), *p* = 0.03. Fisher’s *z* = −0.56	*R* = −0.45 (−0.79 - -0.06), *p* = 0.05. Fisher’s *z* = −0.48
Movement space volume	*R* = −0.53 (−0.84 - -0.16), *p* = 0.02. Fisher’s *z* = −0.6	*R* = −0.59 (−0.86 - -0.23), *p* = 0.007. Fisher’s *z* = −0.68	*R* = −0.46 (−0.73 - -0.17), *p* = 0.05. Fisher’s *z* = −0.5

Values in brackets are the 95% CI of the correlation coefficient.

**Figure 5:**
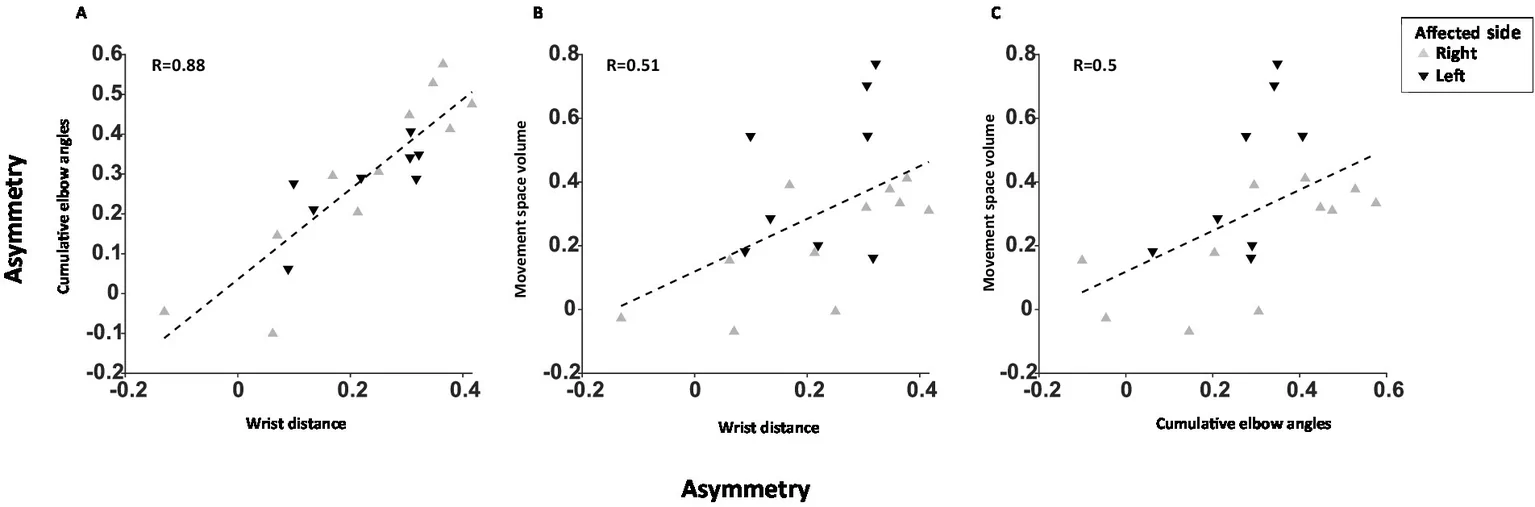
Inter-correlations between asymmetry of elbow angle and wrist distance **(A)**, space volume and wrist distance **(B)**, and space volume and elbow angles **(C)**.

Finally, to assess the relationships between the three activity measures themselves, we computed their inter-correlations. All measures were correlated (R > 0.5, *p* < 0.03; Figure 5). This convergence indicates that the measures capture related aspects of UL use, with some redundancy among them.

## Discussion

In this investigation, we developed a method for monitoring the daily use of the paretic arm in individuals in the subacute stage after stroke. The results demonstrated that IMU-derived kinematic measures of daily UL use can reliably distinguish between paretic and non-paretic arms. All three kinematic measures—wrist path length, cumulative elbow angle, and movement space volume—were significantly reduced in the paretic arm, and these reductions were consistent across participants. Moreover, activity measures based on these measures showed associations with established clinical assessments, particularly the ARAT, with cumulative elbow angle demonstrating the strongest relationship. Collectively, these findings support the feasibility of this approach, and the utility of the IMU-based measures in monitoring and quantifying paretic arm use in PwS during their daily use (activity performance).

Importantly, these objective measures did not merely detect reduced use of the affected arm; they also demonstrated significant associations with clinical impairment scores, indicating that motor impairment influences daily arm use. Kinematic measures correlated most strongly with ARAT scores and, to a lesser extent, with FMA and grip strength. This pattern supports the construct validity of the kinematic measures, suggesting that they capture aspects of UL function that are aligned with clinical evaluation while also offering additional ecological insight into real-world use. Our results are consistent with previous studies showing a correlation between accelerometer-derived asymmetry measurements of intensity and duration of arm movements and motor impairment in PwS (4, 11).

Despite these associations, a substantial portion of the variance in clinical outcomes remained unexplained by the kinematic measures. This discrepancy likely reflects the essential distinction between capacity and performance: clinical tests evaluate what a PwS can (or cannot) do in a structured, supportive environment, whereas participation measures reflect what the PwS do during everyday life. Patients may display relatively preserved ability during clinical testing yet limit spontaneous use of the affected arm in daily contexts due to learned non-use, fear of failure, frustration, or reliance on compensatory strategies (9). In fact, using the affected arm in unimanual tasks is most likely slower, effortful and time consuming than using the unaffected arm, stressing the importance of patients’ engagement in their rehabilitation process even after-hours.

Conversely, some individuals may engage their impaired arm more actively than predicted by their clinical scores, possibly reflecting personal motivation or adaptive rehabilitation efforts (engagement), or preference of using their dominant arm despite its relative greater impairment. Thus, the unexplained variance highlights the complementary nature of these kinematic measures, demonstrating their potential to reveal how PwS respond to and cope with their impairment outside the clinical setting. Recognizing this distinction is crucial, as clinical recovery should not be assumed to translate automatically into functional, real-world use of the affected arm (19).

The present findings underscore that measuring daily performance rather than capacity alone - provides clinically valuable and complementary information about stroke recovery. While clinical assessments remain essential for characterizing impairment, they do not capture the extent to which PwS spontaneously incorporate their affected limb into daily routines. Objective activity monitoring can reveal discrepancies between capacity and actual use, which may otherwise go unnoticed. Such discrepancies may indicate learned non-use or maladaptive compensations that require targeted intervention. Furthermore, monitoring patterns of UL use can offer sensitive indicators of functional recovery, identifying early behavioral changes that are not reflected in structured clinical tests. Because meaningful rehabilitation outcomes ultimately depend on reintegration of the impaired arm into everyday activities, activity-based measures represent a critical dimension of evaluation. Future research should continue to explore UL use as both an outcome measure and a target for interventions.

The interpretation of daily activity measures should also consider the broader challenge of how real-world upper-limb performance is quantified. Previous work has argued that upper-limb functioning in daily life is multidimensional and includes complementary domains such as amount of use, limb preference, movement intensity, and movement quality (20, 21). Within this framework, the three measures examined here capture different aspects of performance: wrist path length reflects overall movement magnitude, cumulative elbow angle reflects the amount of joint-specific movement, and movement space volume reflects the spatial extent of arm use. Although these measures were correlated, their incomplete overlap suggests that no single metric fully characterizes daily upper-limb performance. An additional challenge concerns movement quality. While recent studies have shown that wearable sensors can quantify movement quality during standardized laboratory tasks (22), it remains unclear how movement quality should be defined and interpreted during unconstrained daily-life activities. Consequently, current approaches to monitoring real-world arm use continue to focus primarily on the quantity and intensity of movement rather than on movement quality itself.

A further important observation concerns the overlap and differences between the three IMU-derived kinematic measures. Wrist path length and cumulative elbow angle were highly correlated, with elbow angle explaining up to 77% of the variance in wrist distance. This strong association suggests that both measures reflect similar aspects of UL use, raising the question of whether both are necessary in future applications. By contrast, movement space volume showed weaker correlations with the other two measures, indicating that it captures complementary information related to the spatial extent rather than the magnitude of arm movement. Thus, cumulative elbow angle may serve as the most efficient single measure due to its strong discriminative power and close alignment with clinical outcomes, whereas movement space volume retains value as an independent descriptor of arm use. These results suggest that future implementations may benefit from feature selection process aiming to balance sensitivity, interpretability, and ecological validity.

Several limitations should be considered when interpreting these findings. First, the IMU-derived measures capture general UL movement and do not inherently distinguish between intentional, goal-directed actions and incidental or passive motion. Although asymmetry indices partially mitigate this issue by emphasizing the difference between the two arms, they cannot fully resolve ambiguity regarding the functional meaning of recorded movements. Second, daily activity levels may have been influenced by rehabilitation schedules and therapeutic content, which could introduce variability unrelated to impairment or spontaneous use. Third, the current setup required five sensors to reconstruct UL kinematics, a configuration that is feasible in research contexts but may be too complex for widespread clinical use. An additional methodological limitation concerns the accumulation of orientation-estimation noise in the derived cumulative measures. Small orientation fluctuations may have accumulated over the prolonged recording period and inflated the absolute magnitude of these measures.

Future development should aim to simplify sensor placement, reduce processing demands and account for drifts. Addressing these limitations will be important to enable large-scale implementation of IMU-based activity monitoring in rehabilitation practice.

## Conclusion

This pilot study shows that wearable IMU-derived kinematic measures can quantify daily upper-limb use after stroke, distinguish paretic from non-paretic arm use and are correlated with clinical assessments, particularly the ARAT. By capturing everyday performance rather than clinical capacity alone, these measures reveal discrepancies consistent with learned non-use and compensatory behavior, offering valuable ecological insights that conventional tests may overlook. Although some redundancy exists among the metrics and the multi-sensor configuration may limit immediate clinical adoption, the approach is feasible and highlights the potential for streamlined sensor systems and refined activity measures in future work. Further studies should validate these findings in larger cohorts and explore how activity-based metrics can guide interventions aimed at enhancing real-world engagement of the affected limb.

## Data Availability

The raw data supporting the conclusions of this article will be made available by the authors, without undue reservation.
